# Improved accuracy in colorectal cancer tissue decomposition through refinement of established deep learning solutions

**DOI:** 10.1038/s41598-023-42357-x

**Published:** 2023-09-23

**Authors:** Fabi Prezja, Sami Äyrämö, Ilkka Pölönen, Timo Ojala, Suvi Lahtinen, Pekka Ruusuvuori, Teijo Kuopio

**Affiliations:** 1 Faculty of Information Technology, University of Jyväskylä, Jyväskylä, 40014 Finland; 2 Digital Health Intelligence Laboratory, University of Jyväskylä, Jyväskylä, 40014 Finland; 3 Spectral Imaging Laboratory, University of Jyväskylä, Jyväskylä, 40014 Finland; 4 Department of Biological and Environmental Science, Faculty of Mathematics and Science, University of Jyväskylä, Jyväskylä, 40014 Finland; 5 Institute of Biomedicine, Cancer Research Unit, University of Turku, Turku, 20014 Finland; 6 FICAN West Cancer Centre, Turku University Hospital, Turku, 20521 Finland; 7 Department of Education and Research, Hospital Nova of Central Finland, Jyväskylä, 40620 Finland; 8 Department of Biological and Environmental Science, University of Jyväskylä, Jyväskylä, 40014 Finland; 9 Department of Pathology, Hospital Nova of Central Finland, Jyväskylä, 40620 Finland

**Keywords:** Colorectal cancer, Machine learning

## Abstract

Hematoxylin and eosin-stained biopsy slides are regularly available for colorectal cancer patients. These slides are often not used to define objective biomarkers for patient stratification and treatment selection. Standard biomarkers often pertain to costly and slow genetic tests. However, recent work has shown that relevant biomarkers can be extracted from these images using convolutional neural networks (CNNs). The CNN-based biomarkers predicted colorectal cancer patient outcomes comparably to gold standards. Extracting CNN-biomarkers is fast, automatic, and of minimal cost. CNN-based biomarkers rely on the ability of CNNs to recognize distinct tissue types from microscope whole slide images. The quality of these biomarkers (coined ‘Deep Stroma’) depends on the accuracy of CNNs in decomposing all relevant tissue classes. Improving tissue decomposition accuracy is essential for improving the prognostic potential of CNN-biomarkers. In this study, we implemented a novel training strategy to refine an established CNN model, which then surpassed all previous solutions . We obtained a 95.6% average accuracy in the external test set and 99.5% in the internal test set. Our approach reduced errors in biomarker-relevant classes, such as Lymphocytes, and was the first to include interpretability methods. These methods were used to better apprehend our model’s limitations and capabilities.

## Introduction

Cancer is the term used for a group of diseases that manifest as malignant tumors in any part of the body. Tumors related to cancer are characterized by the rapid growth of cells that extend beyond their normal boundaries. These cells can then metastasize to other parts of the body, effectively spreading the cancer. Metastasis is the primary cause of death due to cancer^1^. According to the WHO^2^, cancer is a leading cause of death worldwide. One in six deaths is attributed to cancer, amounting to approximately 10 million deaths in 2020^2^. The most common sites for cancer to first appear are the breast, lung, colon, and prostate.

Colorectal Cancer (CRC) is the third most common form of cancer and the second deadliest^3^. According to the American Cancer Society, 56% of patients diagnosed are at a stage where the primary cancer has begun to metastasize^4,5^. Early diagnosis and treatment remain of paramount importance^6^. Advancements in fields such as machine vision have substantially improved automatic cancer classification^7–9^. These improvements have been achieved using deep neural networks^10^ with millions of parameters optimized for diagnostic or prognostic purposes^11^. Despite the impressive performance of deep learning, medical experts still need to examine and analyze biopsied tissue samples to confirm diagnosis and tumor staging. The tissue is typically stained with Hematoxylin and Eosin (H&E) to reveal salient histopathological features. Hematoxylin stains histological cell nuclei a purple-blue hue, while eosin stains the cytoplasm and extracellular matrices a pink-red hue.

CRC patients are stratified into different groups to determine personalized treatment and surveillance. These groups typically relate to prognostic clinical outcomes and tumor genetics. To determine these groupings, quantitative biomarkers, clinical data, histopathological analysis of the tumor tissue, and molecular pathology of the tumor cells are used. The biomarkers generally derive from molecular and genetic tests^12–15^. Recent insights into tumor immunology have shown that the tumor microenvironment plays a critical role in tumor development. Therefore, searching for new prognostic and predictive biomarkers that efficiently characterize tumor features is essential.

The first deep learning-based quantitative biomarkers extracted from H&E-stained whole slide images were recently introduced^7,9,16–19^. Kather et al.^7^ presented the first biomarker for CRC stages III and IV that relied on deep learning. This new prognostic biomarker exhibited performance comparable to the current gold standards^20,21^ for determining CRC outcomes. Moreover, the new biomarker could be generated automatically from images with minimal time and financial expenditure.

In their pioneering study, Kather et al.^7^ utilized convolutional neural networks (CNNs)^22^ to learn visual features. CNNs, which are the gold standard in Deep Learning , have been responsible for significant advancements in computer vision. These networks were employed to detect the presence of nine tissue classes from H&E-stained whole slide images^23^. The identified classes were: (1) adipose tissue; (2) background; (3) debris; (4) lymphocyte; (5) mucus; (6) smooth muscle; (7) normal colon mucosa ; (8) cancer-associated stroma; and (9) CRC epithelium. This seminal study achieved 94.3% accuracy across all nine classes in their external testing data. After the classification, the authors combined the output layer neuron activations into a single weighted score , termed ‘Deep Stroma’. This new prognostic CNN-biomarker was subsequently tested for outcome prediction in new patient cohorts. It was found that the Deep Stroma score was a significant prognostic factor, especially in patients with advanced tumor stages (UICC 4). The authors compared the Deep Stroma score against the gold standard of prognostic assessments, which include manual pathologist annotation of the stromal component^20^ and the gene expression signatures CAFs^21^. The results showed that the new CNN-biomarker was highly prognostic in all tumor stages, whereas the pathologist’s annotations and CAFs score were not. This landmark study provided evidence for the efficacy of the new CNN-biomarker and introduced a system that can be employed to detect CRC and other histological components regardless of CRC outcome prediction.

With a 94.3% classification accuracy among all nine classes, the original study^7^ demonstrated that the output neuron activations from the trained model could be used to develop an effective prognostic biomarker for CRC patient outcomes. The newly developed CNN-biomarker depended solely on the visual accuracy of the underlying deep learning system. The overall accuracy of such a system directly influences the relevance and precision of the output neuron activations. In turn, with accurate output neuron activations, the relevance of the new prognostic CNN-biomarker can be enhanced. Subsequently, other studies^24–33^ attempted to improve the underlying system’s accuracy, although often without the capacity to produce the new CNN-biomarker due to incompatible output specifications (i.e., not using output neuron activations) or validation flaws. In this study, we introduced an updated system built upon the foundation of the original architecture^7^, positioning it as an in-place upgrade. Moreover, leveraging our model and block freezing search training strategy, we surpassed the classification accuracy of both the original and all preceding studies. In our final phase, we employed interpretability techniques to dissect and gain deeper insights into the model’s behavior. Our approach aligned with typical experimental workflows in the field^34^.

## Methods

**Figure 1:**
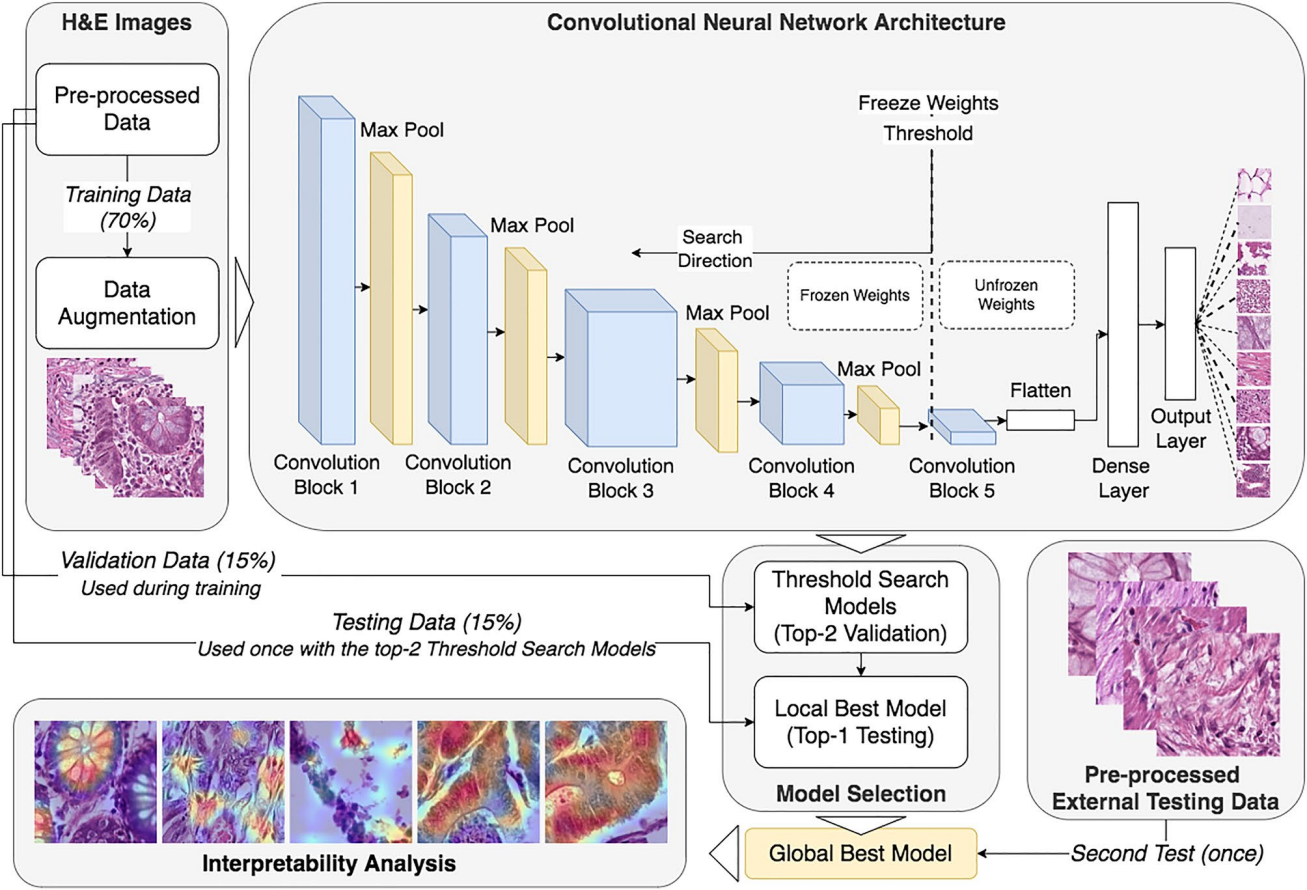
Methodological pipeline for obtaining the best deep learning model.

Figure 1 displays the methodological pipeline employed to obtain the best trained model. In accordance with the figure, we start by describing data acquisition, preprocessing, data augmentation, and neural network architecture design. Finally, we elaborate on training parameters, grid-search parameterization , and interpretability methods.

### Data acquisition and pre-processing

**Figure 2:**
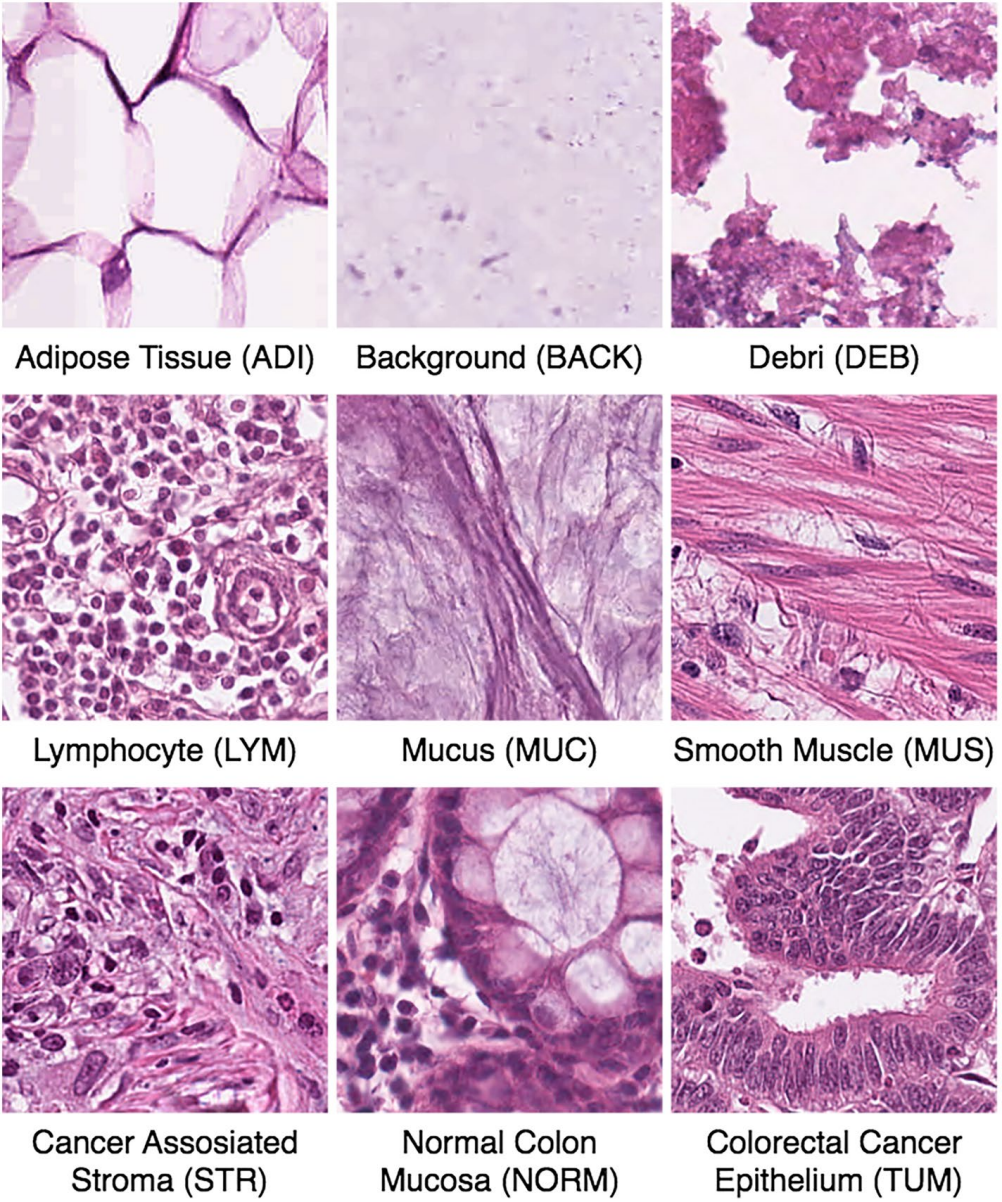
Image tile examples for each class in the training data.

We used the original data specifications as provided by Kather et al.^7^. The dataset consisted of H&E-stained tissue slides from human cancer. These slides were cropped into 224 × 224 pixel tiles and normalized using the Macenko technique^35^. The data^36^ included 86 tissue slides from the NCT (National Center for Tumor Diseases, Heidelberg, Germany) bio-bank and the UMM (University Medical Center Mannheim, Mannheim, Germany) pathology archive. The total dataset comprised 100,000 non-overlapping image patches. These patches were approximately evenly distributed into the following nine classes: (1) adipose tissue (ADI); (2) background (BACK); (3) debris (DEB); (4) lymphocyte (LYM); (5) mucus (MUC); (6) smooth muscle (MUS); (7) normal colon mucosa (NORM); (8) cancer-associated stroma (STR); and (9) CRC Epithelium (TUM) . Figure 2 displays nine image tiles, one for each tissue class. The CRC epithelium was sourced solely from human CRC samples, both primary and metastatic. Although normal tissue like smooth muscle and adipose tissue were primarily derived from CRC surgical samples, they were also sourced from gastrectomy samples (including upper gastrointestinal smooth muscle) to enhance the diversity of the training set.

**Figure 3:**
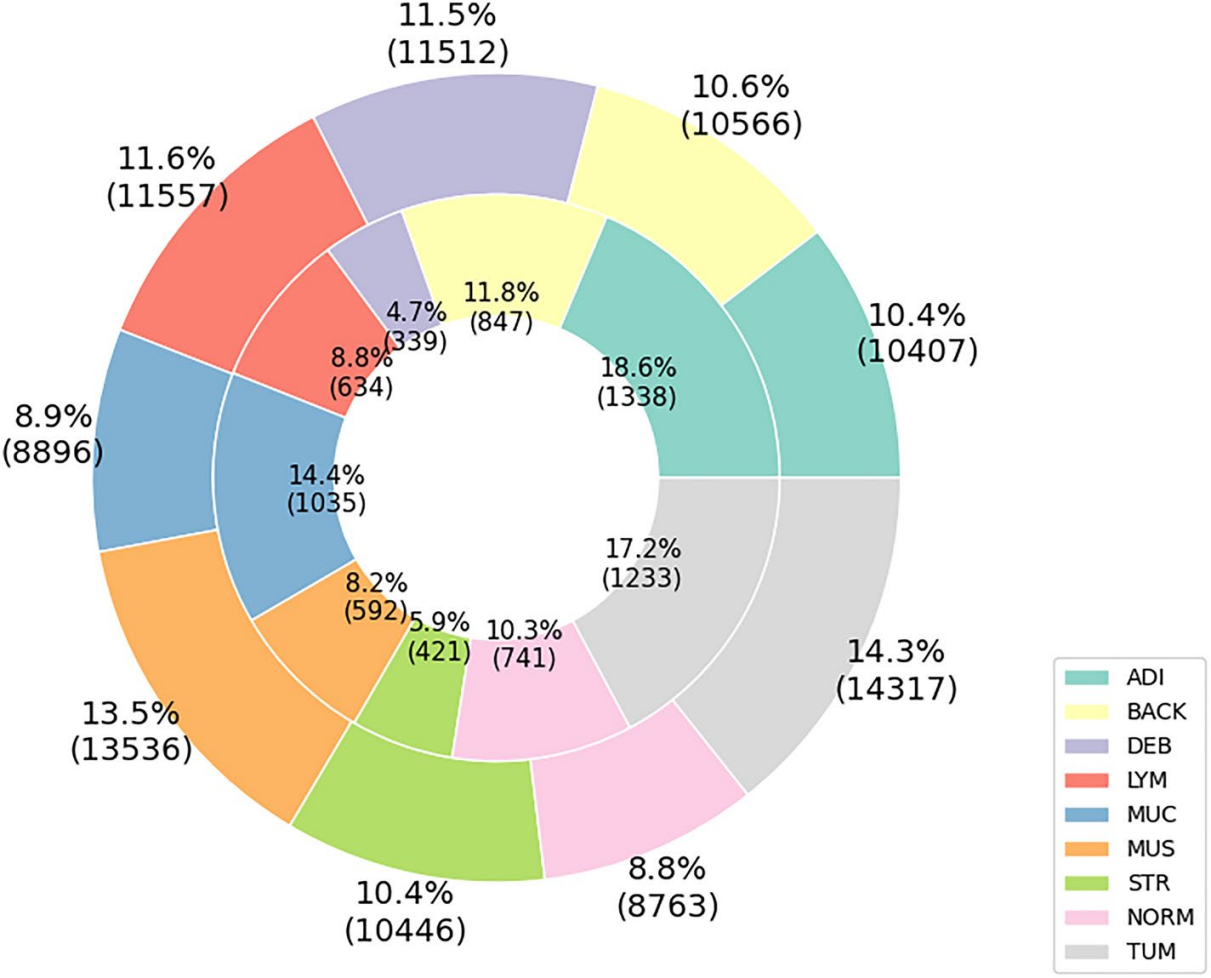
Training (pre-split) and external-testing data details. The outer bar chart displays the total amount of training data in percentages, with the raw number of image tiles shown in parentheses. The inner bar chart follows the same format but for the external testing data.

The data were split into three parts (stratified): a training set, a validation set, and a testing set. These sets contained 69,996, 14,995, and 15,009 images, respectively. The image distribution ratio was 70% of the original data for training, 15% for validation, and another 15% for testing. We also employed the external testing set used in the original work by Kather et al.^7^. The external testing set comprised 25 CRC H&E slides from the NCT biobank, with 7180 image patches, code-named (CRC-VAL-HE-7K)^36^. Figure 3 displays the number of images in each class for the training and external testing data.

In Kather’s study^7^, pure texture regions were manually delineated from 86 CRC slides to compose the initial dataset. Additionally, certain classes underwent augmentation with added samples sourced from externally designated slides. Without patient identifiers, the partitioning of data was carried out randomly, reserving unique patient-level slides exclusively for the external test set. Given these constraints, our intent to stratify splits at the patient or slide level was not feasible.

All images in all the sets used default VGG19^37^ input standardization as follows; For image ${\varvec{I}}$ in data partition set $train=\{{\varvec{I}}, \ldots ,{\varvec{I}}_n\}$ we obtain channel vector ${\varvec{I}}_c$ of $m\times n$ dimensions. Using the channel cumulative distribution function (cdf) and pixel value *v*, we obtain new pixel value *h*(*v*) for that channel by:

1 $$\begin{aligned} h(v) = \text{cdf}(v) - \text{cdf}_{\mu_c} \end{aligned}$$

where $\text{cdf}_{\mu_c}$ is the average value of channel *c* across all images in the training set. The operation repeats for each R, G, B channel and each dataset partition.

### Data augmentation

In deep learning, data augmentation^38^ serves as a technique to artificially enhance the diversity of training images. By transforming images randomly prior to their inclusion in the training phase, a more varied dataset can be emulated, as exemplified by random image rotation. The incorporation of multiple augmentation methods can lead to a combinatorial increase in potential variations. In our study, we used six data augmentation methods sourced from the Keras python repository^39^. It’s worth noting that, unlike the original study which only used random horizontal and vertical flips, our approach added several other affine transformations, further enhancing the dataset’s diversity. Details about our data augmentation approaches and their configurations are provided in the Supplementary file (Table S1). The exact configuration is also available under the ’advanced’ augmentation preset in the Deep Fast Vision library^40^ .

### Convolutional neural networks

Convolutional neural networks (CNNs)^22^ are foundational to the recent deep learning revolution^10^. CNNs are a type of neural network primarily used in computer vision. These networks employ the convolution operation between the input and a filter-kernel. Filters slide across the input to highlight features , producing a response known as a feature map. Various feature maps combine to produce higher-level feature maps , corresponding to more complex concepts. Formally^41^, for an image ${\varvec{I}}$ of $m\times n$ dimensions and filter-kernel ${\varvec{K}}$ of $q\times r$ dimensions, we can obtain feature map ${\varvec{F}}$ by convolution across the two axes *m*, *n* with kernel ${\varvec{K}}$ as:

2 $$\begin{aligned} {\varvec{F}}(m,n)=\sum _{q}\sum _{r}{\varvec{I}}(m,n){\varvec{K}}(m-q,n-r) \end{aligned}$$

Typically, the values of the feature map are filtered with an activation function. The activation function’s role is to remap values across a given function. For instance, the rectified linear unit activation function (ReLu)^42^ zeros out negative values. This approach offers computational efficiency by replacing redundant values with zero. For any feature map value *x*, the ReLu activation is defined as:

3 $$\begin{aligned} f(x)= \max(0,x) \end{aligned}$$

In addition to the activation function operation, the max pooling operation is also frequently used. Max pooling down-samples the convolution result, so cascades of max pooling and convolution lead to an ever-decreasing number of features. For image ${\varvec{I}}$ of $m\times n$ dimensions, the max pooled value ${l(m_I)}$ given dimension *m* can be simply defined as follows:

4 $$\begin{aligned} l(m_I)= \lfloor \frac{{m_I}-p}{s}\rfloor +1 \end{aligned}$$

where $m_I$ is only dimension *m* from image ${\varvec{I}}$, *p* is the pooling window size and *s* is the stride value.

**Figure 4:**
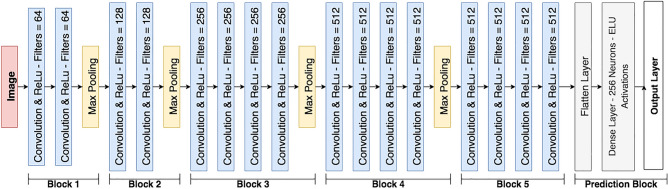
The CNN architecture used in this study.

We utilized the VGG19^37^ CNN architecture as the foundation for our neural network design. Kather et al.^17^ evaluated various unaltered architectures and demonstrated that the original VGG performed the best in these experiments. The CNN was pre-trained with the ImageNet^43^ dataset , which contains 14 million images distributed across 20,000 categories. A network pre-trained with ImageNet weights frequently serves as the starting point for many deep transfer learning vision classifiers. Our VGG19 variant incorporated all five VGG19 convolutional blocks, while the classification head was simplified to 256 units. The dense layer employed exponential linear unit^44^ (ELU) activations, while the output layer used softmax^45^ activations. Each convolutional block consisted of convolutional layers with ReLu activations followed by a max pooling layer. Figure 4 showcases the utilized architecture.

In training our neural network, we employed the Adam optimizer^46^ with parameters $lr=0.00002$, $\beta _1 = 0.9$, and $\beta _2=0.999$ . Here, *lr* denotes the learning rate , while $\beta _1$ and $\beta _2$ represent the decay rates of the first and second momentums, respectively. To determine the baseline learning rate, before initiating the weight freeze, we conducted a simple grid search. We varied the learning rate by an order of magnitude (i.e., 0.0002, 0.00002, and 0.000002), monitoring only the validation accuracy, which led us to our chosen value. We highlight the betas (default values) because users not using the Tensorflow library might encounter different default values. For our training process, we utilized the categorical cross-entropy loss and trained for 22 epochs with batches of 128 images. All weights that weren’t pre-trained were initialized using the HE normal distribution^47^ . The input was specified as $224 (\text {W}) \times 224 (\text {H}) \times 3 (\text {RGB})$ .

### Sequential weight-freeze search

The most effective model in this study was identified by determining the ideal threshold for freezing weights between the convolution blocks. We initialized the five VGG convolutional blocks in our model using pre-trained weights from ImageNet training. During transfer learning, it ’s common practice to freeze some of these weights, preventing them from being modified during subsequent training. Thus, the learned features remain unchanged.

Our sequential approach began by freezing the weights of all blocks. We recorded the performance of the model in this state. Then, we progressively unfroze the weights of blocks, starting with the fifth VGG convolutional block. After each block was unfrozen, we trained the model and recorded its performance. This process continued until only the first block remained frozen. The specific parameters of this sequential weight-freezing search are detailed in Table 1. It’s essential to note that this weight freezing strategy applied only to the VGG blocks; the dense and output layers remained unaffected. To determine the optimal configuration, we considered the two configurations with the highest validation accuracies. These configurations were then evaluated using the designated test set. The best-performing configuration from this internal testing was subsequently validated using the external test set .

**Table 1:** Sequential weight-freeze search for VGG19.

Search step	Conv-Block 1	Conv-block 2	Conv-block 3	Conv-block 4	Conv-block 5
1	Fixed	Fixed	Fixed	Fixed	Fixed
2	Fixed	Fixed	Fixed	Fixed	Trainable
3	Fixed	Fixed	Fixed	Trainable	Trainable
4	Fixed	Fixed	Trainable	Trainable	Trainable
5	Fixed	Trainable	Trainable	Trainable	Trainable

The table illustrates the stages of the Sequential Weight-Freeze Search, beginning with Convolutional (conv) Block 5 and progressing to Convolutional Block 1. “Trainable” labels signify weights that could be adjusted (’unfrozen’), while “Fixed’ labels denote weights that remained immutable.

### Model interpretability

Convolutional neural networks have significantly impacted computer vision in medicine^48–50^. Unfortunately, with the increase in neural network complexity comes difficulty in interpreting the clear etiologies of predictions, especially on a per-instance basis. Consequently, many such systems are often referred to as ’black boxes’. However, interpretability is essential to foster trust in intelligent systems^51^. An interpretable system offers the potential for better societal integration and expert intervention upon systematic errors. The Grad-CAM algorithm^52^ enabled us to perform an interpretability analysis directly from our vision system, thereby reducing the ’black box’ effect. Based on the original CAM framework^53^, Grad-CAM produces spatial activation maps. These maps can highlight regions within a given image that contributed positively to a specific prediction. Grad-CAM can be calculated as follows:

5 $$\begin{aligned} a _{k}^{c} = \frac{1}{Z} \sum _{i} \sum _{j} \frac{\partial y ^{c}}{\partial \Theta _{ij}^{k}} \end{aligned}$$

where $a _{k}^{c}$ is neuron importance weights of feature map *k* for class c, $\frac{\partial y ^{c}}{\partial \Theta _{ij}^{k}}$ is the partial derivative of the final layer prediction for class $c y ^{c}$ with respect to the last convolutional layer *k* th feature map $\Theta _{ij}^{k}$. In addition, *Z* is the total pixels, and *i*, *j* the indexes for each element within feature map *k*. Given the ReLU activation, we can obtain the Grad-CAM output as:

6 $$\begin{aligned} L_{Grad-CAM}^{c} = ReLU (\sum _{k} a _{k}^{c} \Theta ^{k}) \end{aligned}$$

where $\Theta ^{k}$ is the feature map k given by last the convolutional layer, averaged spatially. The $L_{Grad-CAM}^{c}$ is the final spatial activation map produced by Grad-CAM. In our experiment, Grad-CAM produced activation maps of the final convolutional layer (block 5) . This approach allowed us to conduct interpretability analyses on individual image tiles. Preliminary analysis revealed that the last convolutional layer in block 5 appeared to adeptly localize complex high-level features. For instance, regions corresponding to tumors and other pathologically significant structures suggested that the layer learned to identify and represent complex features in its processing. A collaborating senior medical expert histopathologist from Hospital Nova in the Central Finland Healthcare Region analyzed, reviewed, and detailed the Grad-CAM results.

## Results

### Classification

**Figure 5:**
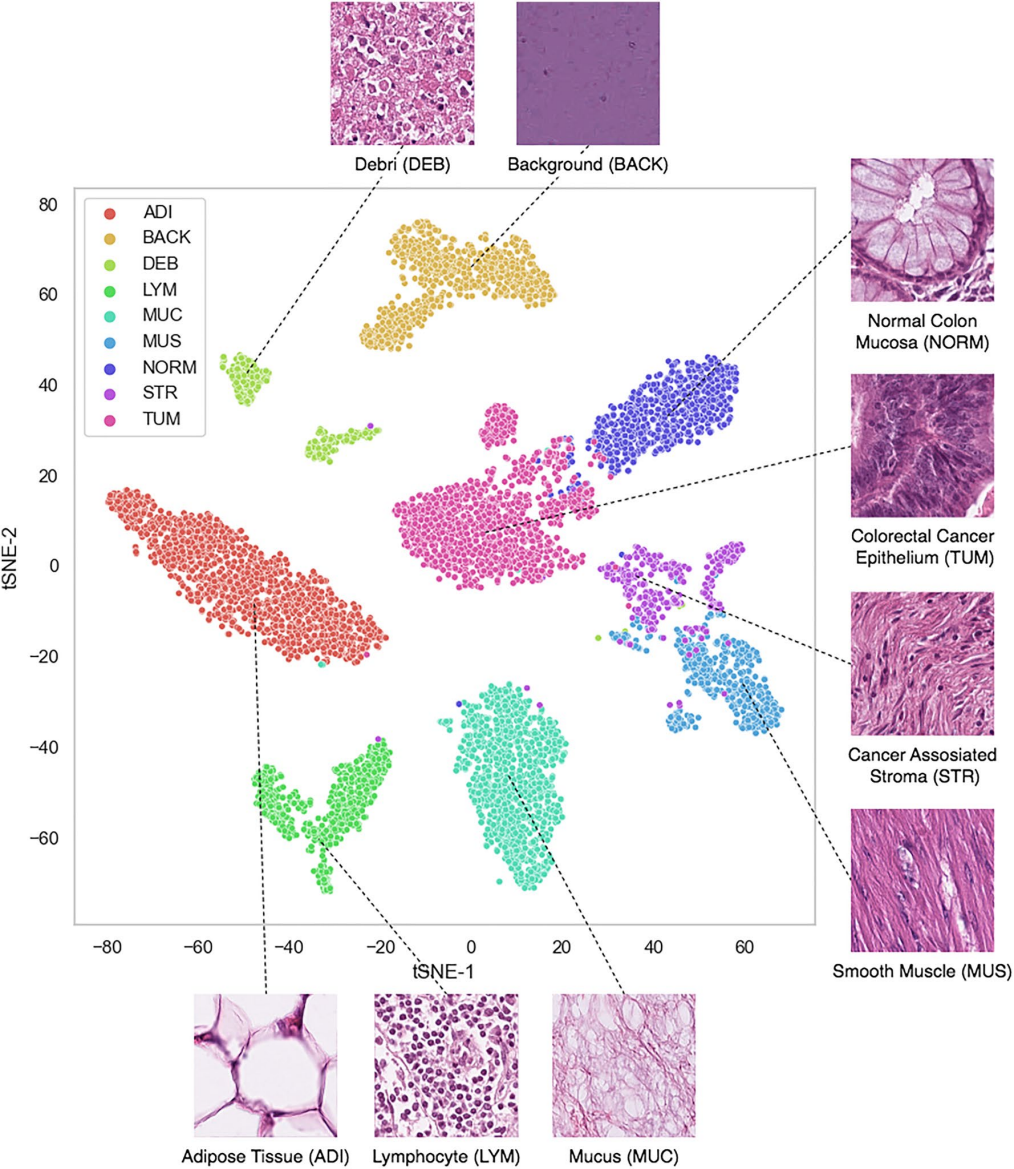
Scatterplot of the t.SNE projection from the best-trained model. The projection was calculated from the post-flatten and pre-output dense layer. All data points depicted belong to the external testing set.

**Figure 6:**
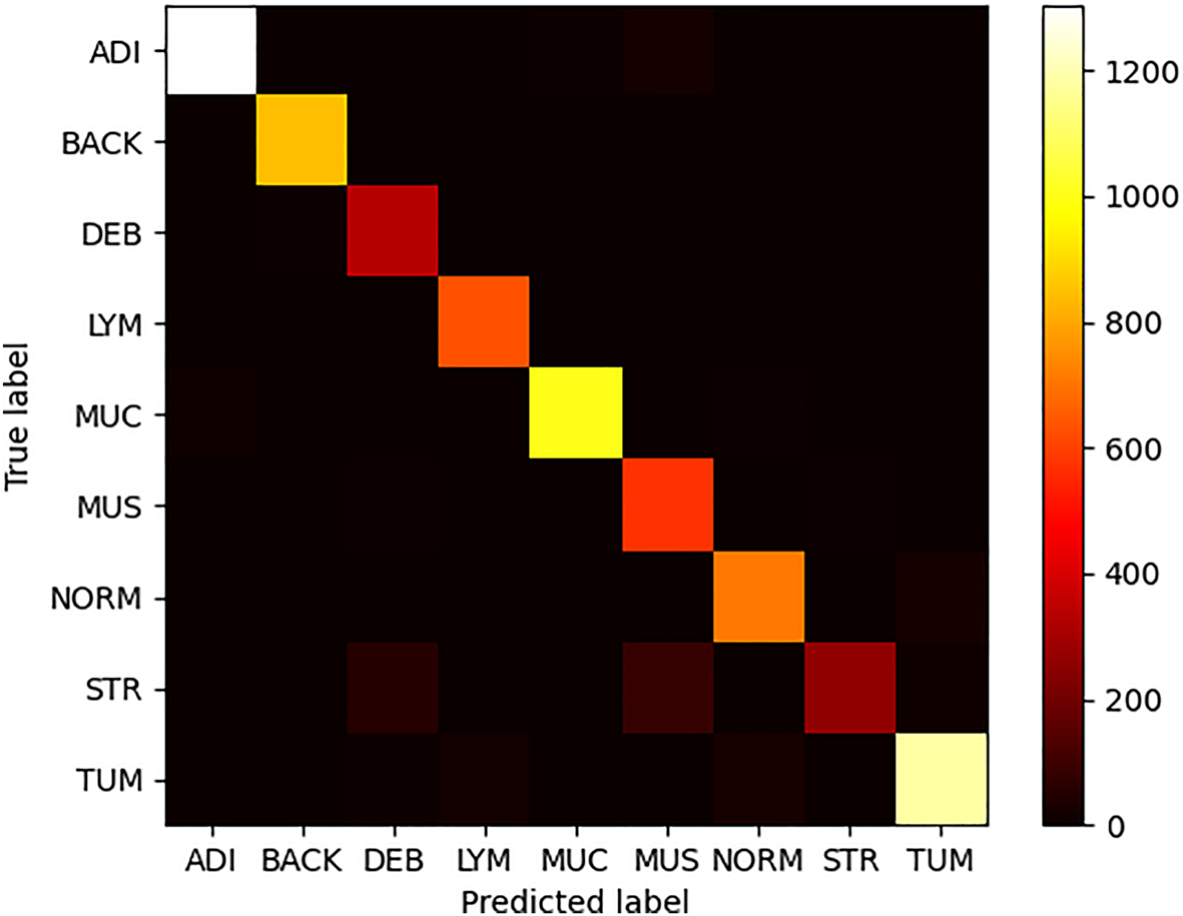
External testing set confusion matrix with respect to image tiles and classes. This figure has an identical color format as in Kather et al.^7^ and can be compared directly.

In this study, we built upon the best architecture identified and validated by Kather et al.^7^, advancing not only beyond their groundwork but also outperforming the results of subsequent studies that followed their seminal work. Our approach involved refining the original architecture and incorporating a block freezing search, serving as the key technique for hyper-parameter optimization. Table 2 showcases the results of that search, highlighting the best-found model. The external testing data further evaluated the best model (Frozen on CV Block 2). We achieved 99.5% accuracy on the internal testing set and 95.6% on the external test set. We observed a trend of increasing accuracy scores the further we unfroze weights. We focused on the best-found model by generating its confusion matrix and t-distributed stochastic neighbor embedding^54^ (t-SNE) plot. As shown in Fig. 5, the separation between the classes was near optimal and not fragmented. The relative distance between the classes was in line with histopathological expectations, i.e., tumor and normal classes being close , and stroma-muscle as well, which further highlighted the potential for misclassification. This potential is also evident from the confusion matrix in Fig. 6, in which we see that most classes were optimally classified. The primary misclassifications occurred between the classes stroma-muscle-debri.

**Table 2:** Accuracy in different locations of the weight-freeze search.

Weights freeze threshold (frozen at and below threshold)	Training accuracy	Validation accuracy	Testing accuracy	External testing accuracy (CRC-VAL-HE-7K)
Convolutional block 1	99.4%	99.3%	99.4%	–
Convolutional bblock 2	99.4%	99.3%	99.5%	95.6%
Convolutional block 3	99.3%	99.5%	99.3%	–
Convolutional block 4	98.6%	98.9%	–	–
Convolutional block 5	93.6%	95.1%	–	–

Table 3 shows the accuracy scores obtained by all studies employing the original and external testing data. Our model outperformed all current studies and was the only one based on the original architecture search findings by Kather et al.^7^ . Additionally , our study was the only one with complete error reporting, from training to external testing. Other than the original study, studies^29,30^ that did not use valid validation approaches, such as no validation and testing data for detecting overfitting, including parameter-hyperparameter search on external testing data are not eligible for comparison. Lastly, instances with few-shot learning and testing^31^ , shuffling of external testing data within training data^32^ , and using external testing as validation and testing with their own testing data^33^ also do not qualify for comparison. Our best-trained model and other related materials can be found under data availability.

**Table 3:** Deep learning accuracy and validation method comparison across all relevant studies.

Study	Training set accuracy (TR)	Validation set accuracy (V)	Testing set accuracy (T)	Validation approach	External testing set accuracy (T2)
Ours	99.4%	99.3%	99.5%	TR-V-T-T2	95.6%
Kather et al.^7^ (2019) (original)	–	–	98.7%	TR-V-T-T2 TR-T2	94.3%
Peng et al.^24^ (2019)	–	–	–	TR-V-T-T2	95%
Qi et al.^25^ (2021)	–	99%	95%	TR-V-T2	95%
Shen et al.^26^ (2022)	–	–	–	TR-V-T2	94.8%
Wang et al.^27^ (2021)	–	–	–	TR-V-T2	94.8%
Yang et al.^28^ (2021)	–	–	–	TR-V-T2	91.1%
Yang et al.^55^ (2021)	–	–	–	TR-V-T2	86.4%

**Figure 7:**
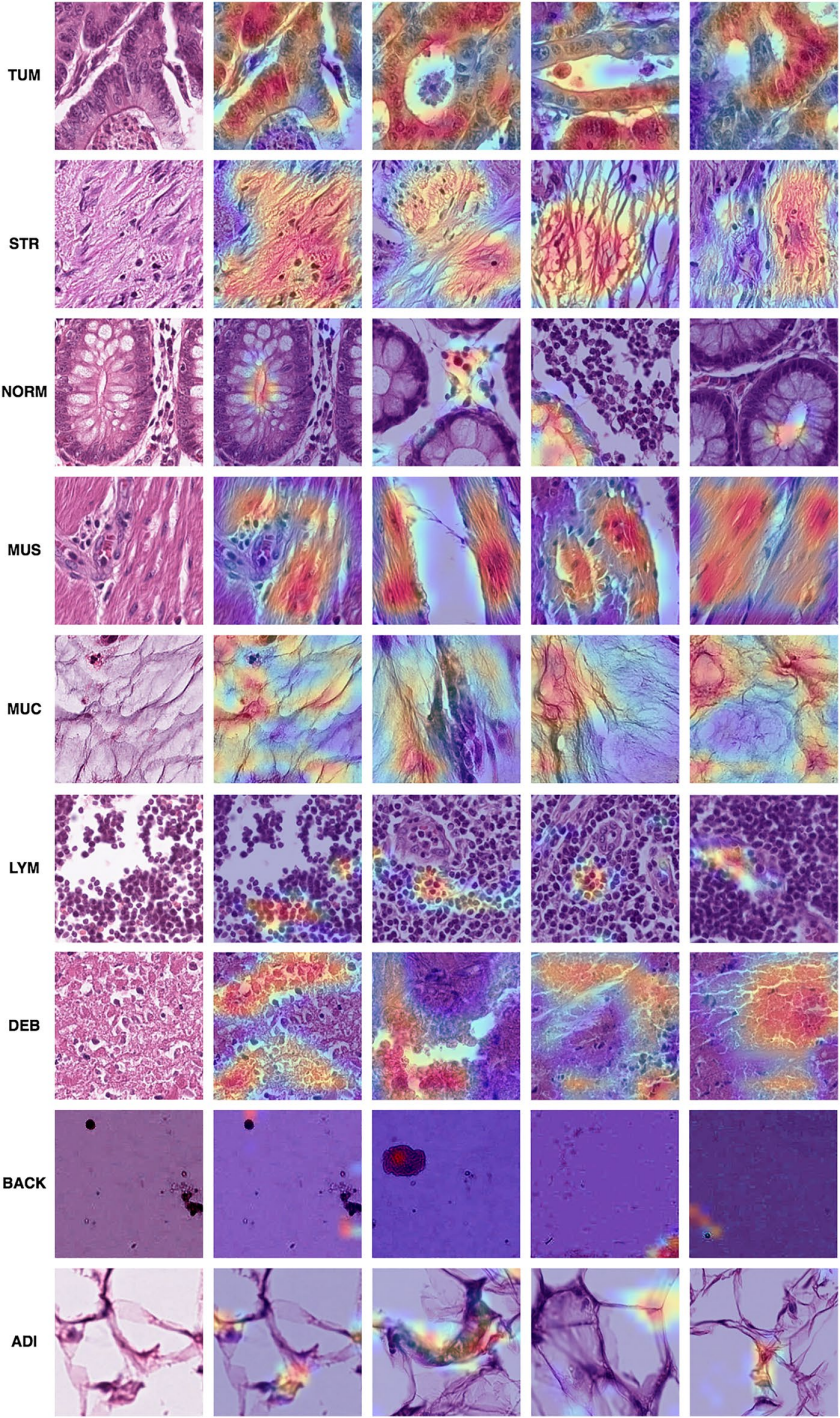
Grad-CAM activations for top-1 tiles in the external testing data. Activations range from blue to red, with red indicating the maximum activation for the class. Only positive activations are shown. All images were correctly classified with over 99% confidence. The first column contains the raw images, corresponding to each image in the second column.

**Figure 8:**
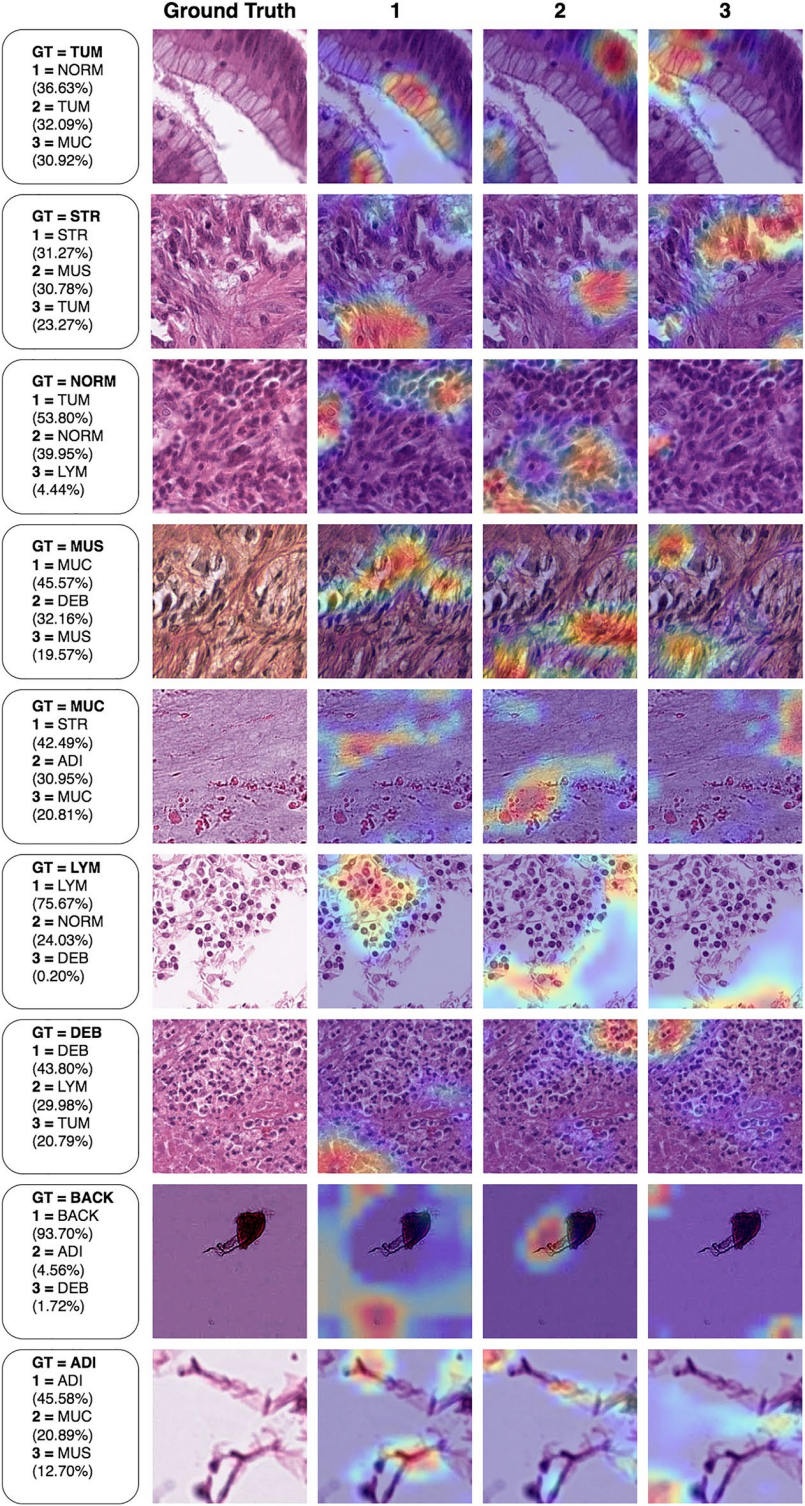
Grad-CAM activations for top confusing tiles in the external testing data. Activations range from blue to red, with red indicating the maximum activation for the class. All activations are positive. Each image has minimal confidence variance across all classes. Each row displays one image, and the annotation box provides information about the predicted classes and their corresponding confidence in descending order. Numeric markers correspond to activations of the predicted classes. “GT” stands for the ground truth class, and the ground truth image is presented in its raw form from the dataset.

Figures 7 and 8 display Grad-CAM activations for the external testing set data. Figure 7 presents activation maps for 36 top-1 predictions , each having over 99% prediction confidence. Figure 8 exhibits top-1 misclassifications with minimal variance in prediction confidence , both within and across all labels. Figure 7 identified the following regions as being pertinent to those predictions : ADI, cell membranes and other cellular structures of adipocytes; BACK, non-specific artifacts; Debri, necrotic material; LYM, small lymphocytes; MUC, mucoid material; MUS, smooth muscle cells; NORM, normal colonic crypts and lamina propria; STR, extracellular collagen fibers; TUM, cancer cells. For the top 1 tiles , we observed correctly localized activations from relevant morphology. This effect was consistent across both homogeneous and heterogeneous tissues, as evidenced in TUM, NORM, MUS, MUC, LYM , and DEB.

In Fig. 8, we observed mostly accurate activation localization . However, for cases such as TUM, NORM, MUS, and MUC, the predicted class was incorrect. In all examples, classifier confidence was low and distributed amongst three classes, except for LYM and BACK. For TUM, we identified highlighted cancer cells (TUM1, TUM2, TUM3), while the background was ignored. Yet, NORM received a 4.54% higher confidence than the true class (TUM). In NORM, no typical epithelial cells of normal mucosa were observed . Lower-density regions (NORM 1, 2, 3) were emphasized . The top class (TUM) held a confidence 13.85% greater than the true class (NORM). In MUS, we recognized autonomic nerve structures (MUS 1, 2, 3). The top predicted class (MUC) was 26% more confident than the true class (MUS). In MUC, we discerned mucoid material and red blood cells. The top prediction (STR) had a confidence level 21.68% higher than the true class.

In Fig. 8, the remaining examples had correct predictions but low confidence. In ADI, low confidence activations for MUC (ADI 2, 3) were localized on cell membranes. The true class (ADI) had a 24.69% higher confidence than the second label (MUC). In BACK, a non-specific artificial structure in the middle of the tile slightly activated the ADI class (BACK 2). The confidence in the true class was 93.70%. In DEB, regions at the top right edge activated LYM (DEB 2), while regions at the top left edge activated TUM (DEB 3). The morphology of the edges varied. DEB 1 and 3 related regions contained acellular necrotic material adjacent to degenerated inflammatory cells. The DEB 2 region contained only degenerated inflammatory cells. The true class was 13.82% more confident than the second label (LYM). In LYM, the second label (NORM) was activated in the border region, which contained cellular elements and no lymphocytes. The true class was 51.64% more confident than the second label (NORM). Lastly, in STR, a small patch of paucicellular fibrous stromal area activated MUS (STR 2), while cancer cells in the cellular region activated TUM (STR 3). The true class (STR) had a 0.49% higher confidence than the second label (MUS).

## Discussion

Our study demonstrated that a weight freeze search on an established VGG model produced a more accurate and effective CRC classifier than before. We surpassed previous approaches in terms of accuracy without increasing architectural complexity. We believed that a direct approach on the original classifier was the best way to maintain relevance to the breakthrough study by Kather 2019^7^ and to make our features directly applicable for further experiments in patient outcome prognostication. The reason for this potential is that only neuron activations of the output layer consisted of the original biomarker basis. The original CNN-biomarker , named ’Deep Stroma’ , incorporated a selection of classes. We not only retained the accuracy in the subset of features used for Deep Stroma but also improved upon the lymphocyte class accuracy needed for its calculation. These outcomes rendered our model an in-place update of the original approach. In this regard , switching the output layer with any other machine learning approach, such as support vector machines^56^, KNN^57^ , etc., would disable the potential use for this purpose, even though it might improve accuracy. We believe a classifier agnostic approach for calculating deep-stroma might be warranted for future work and further improved results.

Regarding augmentation and validation, we found that strong data augmentation effectively combated overfitting given the appropriate validation approach. This was not surprising, as it is a fairly standard approach used in a plethora of classification studies. As seen in Table 3, we observed variation in validation approaches, which in itself constitutes a problem when validation, testing, and external testing sets are absent. The state of validation in this problem would benefit from a uniform approach. Limitation-wise, training such systems requires vast amounts of annotated data; potentially improving such systems would require even more data to be annotated by medical experts. We note that several studies^26,28,29,33^ are not peer-reviewed yet and thus are of limited comparative reliability.

Regarding the choice of classifier and novelty, Kather’s^7^ approach left certain areas unexplored. Notably, the study did not explore the effects of model probability calibration^58^ on the deep stroma score after pinpointing the best-performing classifier. This approach could be particularly significant, considering that deep stroma heavily relies on the probabilities emitted by the output layer, as well as the overall accuracy of the system. It is crucial to note that different vision architectures manifest distinct class probability profiles^58^ (i.e., systematically over or under-confident in predictions), even if performance metrics remain nearly identical. Consequently, the only validated architecture for the advanced stages of outcome prediction with deep stroma was anchored to the intrinsic probability profile of the VGG19 model. Hence, the ramifications of architecture changes on these profiles regarding deep stroma and, by extension, outcome prediction remain uncharted territories. This scenario restricted our purview in exploring novel model architectures. To ensure that our contributions remain germane and maintain as much potential for outcome prediction tasks, we deliberately chose to align with the only validated architecture whose associated probability profile was shown to be effective in the later stages of outcome prediction. This informed decision also steered our focus toward devising the weights-freeze search, prioritizing it over changes to the existing architecture. The points highlighted suggest a new avenue for future research. Specifically, understanding how probability profiles influence deep stroma scores, and consequently, outcome prediction.

Regarding parameter search, the most effective approach for improving accuracy was to search for which block of weights to freeze. To the best of our knowledge, this approach has not been featured elsewhere as a systematic search method. We performed our search linearly and did not incorporate variations of weight freezing between distant blocks. We strongly believe this approach may generalize further and is worthy of further investigation. Limitation-wise, it is worth mentioning that we did not search for any hyperparameter values after the parameter search. In the future, and given more complex search schemes, such as combining weight freezing with Bayesian search methods, we believe that accuracy might improve even further.

Regarding microscope image quality, the quality of slides and the presence of artifacts or pixel noise could play a catalytic role in generating misclassifications. Tiles undergo normalization and contrast enhancement before they enter the classifier; thus, pixel noise or other non-tissue artifacts such as dust or hair might be accentuated and potentially skew results. The ‘Picasso’ effect^59^ can further exacerbate these outcomes in CNNs. Scenarios in which little or no relevant tissue is present in a given tile, and the tile is not assigned as background, could also lead to misclassifications. Although having a background class can help minimize such situations, the effect is partial, and these kinds of mistakes would be expected. The recommendation for future systems would be to introduce pixel noise randomly within the augmentation phase, with replacement. Replacement and randomization are essential; a lack thereof could induce biases and potentially be over-fitted by the classifier. Lastly, the focus factor (‘blur’) can also affect outcomes, especially when paired with pixel noise. In this respect, further augmentation with various blur intensities could be recommended. These recommendations are especially critical since not all patient slides may have the same focus and quality.

With regard to robustness to whole slide image artifacts, in the foundational study that generated the data we utilized, all slides underwent meticulous manual review by the authors. Slides showing tissue folds, torn tissue, or other noticeable artifacts were omitted. However, this systematic exclusion means models, including ours, lack exposure to these challenges. As a result, in scenarios where such artifacts are prevalent, the system may not display the desired invariance. This underscores the importance of training datasets that are representative of the variability and challenges a system might face in its intended application environment. Although affine augmentations and training on a background class can help, these approaches are not replacements for including challenging conditions.

Regarding overall model confusions, when comparing our confusion matrix with the original confusion matrix^17^ (using the same heatmap), we can see an accuracy boost in the lymphocyte (LYM) and background (BACK) classes. This boost is relevant since lymphocyte detection is also part of the Deep Stroma score. In addition, when background and tissue are both present in any given tile, better background accuracy may reduce false positives and provide more robustness against pixel noise. We observed that stroma predictions were similar, but confusions differed. In our model, some stroma patches were confused with muscle and debris , while in the original^7^, there were more confusions between muscle and lymphocytes. When comparing the two models in terms of t-SNE feature space, we found two main differences; in the original model, stroma, mucosa, and lymphocytes were fragmented. Part of the fragmentation was distributed far from most class instances. In our model, no such effect was found; this indicated better separability and subclass cohesion in the projected vector space. Overall, class proximities in the projected space aligned with the initial findings^7^.

Regarding the interpretability results, in Fig. 7, we saw correct top-1 predictions associated with relevant regions in the image tiles. This indicated that the model based its decisions on relevant morphology and higher-level structures. We observed that non-relevant classes were ignored even when they were partially or necessarily present, for example, in the TUM, NORM, MUS, LYM, and ADI examples. Similarly, bottom-1 predictions from Fig. 8 displayed accurate localization but often did not trigger the correct predictions. Upon analyzing these mistaken predictions, we identified a key similarity. Such predictions appeared to feature at least one other class. In TUM, we found both cancer cells and background; in NORM, we observed some lymphocytes but no typical epithelial cells of normal mucosa. The tissue closely resembled stroma (STR) without cancer cells. Since lymphocytes can be present in both normal and cancerous tissue, the top confusion could be partly attributed to the presence of lymphocytes and the absence of typical normal cells. These observations suggest that the annotated ground truth might be mistaken; in MUC, we identified acellular mucoid material and red blood cells. However, the image seems to show a fibrillar arrangement, which might partially explain the top confusion. Fibrillar arrangements and collagen fibers are typically found in stroma; in MUS, we discovered autonomic nerve structures within smooth muscle. Both nerves and mucoid material do not stain intensely; in this context , their presence might account for part of the top confusion. We encountered similar issues for low confidence but correctly predicted examples in the same figure. ADI featured a cropped cellular structure in the bottom-left corner; BACK displayed a non-specific histologic structure in the center of the tile; LYM had some background due to its near-boundary position; Clear etiologies for confused predictions are hard to pinpoint. However, it appears that mixed tissue, combined with other limitations mentioned previously, might be influential and should be taken into account for further analysis.

Regarding image size, in both Figs. 7 and 8, we observed that small regions within tiles often had maximal activations toward a given class. This was not surprising, given that most classes contained varying repetitive morphological structures. The result strongly suggested that the amount of information in each tile often appeared to be more than sufficient. In this regard, the zoom level (0.5 microns per pixel) could be adjusted to produce even more tiles while remaining relevant for explaining each class. However, such zoom adjustments are challenging to estimate for sufficient coverage across all tissue classes. Nonetheless, a model trained in this way might identify annotation outliers and mixed tissue tiles that lead to mixed results. In this regard, we expect that ’confused’ predictions from well-trained and augmented systems could also assist specialists in identifying annotation mistakes, artifacts, or indistinct slide regions.

Regarding the state of the literature, we have observed significant progress made within a relatively short period of time. However, several limitations exist . First, we noticed an inconsistent evaluation approach among various systems. Consistency and best practices in evaluation help minimize biases and allow for direct comparisons. Additionally, metrics such as label noise have not yet been estimated. Label noise estimates could help set the ceiling for future comparisons. We found no discussion on system robustness during or after training, an essential issue in development and testing. Lastly, we did not find any interpretability analysis conducted before this study. We demonstrated that such an analysis could help clarify limitations and indirectly suggest future steps. We strongly recommend future studies consider such an analysis to interpret some black-box behavior, especially given the medically critical nature of these systems.

Overall, this work needs to be clinically validated before routine clinical deployment. We see significant promise both in classifying CRC slides and in terms of potentially improved Deep Stroma scores, which in turn aspire to manifest in better CRC outcome predictions. As part of this study, and in contrast to most related studies, we provide open access to all our models and materials.

### Supplementary Information

Supplementary Table S1.

## Data Availability

The best model, weights, and data generated during the current study are available at Mendeley Data: https://data.mendeley.com/datasets/8wz5dttyyz/1.
