# Supplementary material for: Improved accuracy in colorectal cancer tissue decomposition through refinement of established deep learning solutions
Source: Sci Rep. 2023 Sep 23;13:15879. doi: 10.1038/s41598-023-42357-x (PMC10517936; doi:10.1038/s41598-023-42357-x)
Supplement: Supplementary file 1 — Supplementary Table S1. [file 41598_2023_42357_MOESM1_ESM.pdf]

Supplementary Materials

**Augmentation Method**	**Method Description**	**Training Configuration**
Rotation	Rotates images	Rotates image up to 40 degrees
Width Shift	Shifts image along axes	+- 45 pixels shift along width axes
Height Shift	Shifts image along axes	+- 45 pixels shift along height axes
Shear	Image is distorted ('stretched') along width or height axis	A 0.2 degrees maximum shear angle
Zoom	Zooming within image	Maximum zoom of 20%
Horizontal Flip	Mirror image along orientation	Only horizontal flip

Table S1. Augmentation approaches used for neural network training.
